# Community-Acquired Skin and Soft Tissue Infections: Epidemiology and Management in Patients Presenting to the Emergency Department of a Tertiary Care Hospital

**DOI:** 10.7759/cureus.34379

**Published:** 2023-01-30

**Authors:** Qaidar Alizai, Abdul Haseeb, Sana Hamayun, Shandana Khan, Fawad Ali, Munayal Roghani, Muhammad Awais Khan, Farhan Ullah, Waseem Khan, Nadeem Ijaz

**Affiliations:** 1 Department of Surgery, Hayatabad Medical Complex Peshawar, Peshawar, PAK; 2 Department of Medicine, Khyber Teaching Hospital Medical Teaching Institute (MTI), Peshawar, PAK; 3 Department of Surgery, Khyber Teaching Hospital Medical Teaching Institute (MTI), Peshawar, PAK

**Keywords:** emergency management, skin infections, anti-bacterial agents, emergency medical service, soft tissue infections

## Abstract

Background: Skin and soft tissue infections are one of the most common diseases presenting to the emergency department (ED). There is no study available on the management of Community-Acquired Skin and Soft Tissue Infections (CA-SSTIs) in our population recently. This study aims to describe the frequency and distribution of CA-SSTIs as well as their medical and surgical management among patients presenting to our ED.

Methods: We conducted a descriptive cross-sectional study on patients presenting with CA-SSTIs to the ED of a tertiary care hospital in Peshawar, Pakistan. The primary objective was to estimate the frequency of common CA-SSTIs presenting to the ED and to assess the management of these infections in terms of diagnostic workup and treatment modalities used. The secondary objectives were to study the association of different baseline variables, diagnostic modalities, treatment modalities, and improvement with the surgical procedure performance for these infections. Descriptive statistics were obtained for quantitative variables like age. Frequencies and percentages were derived for categorical variables. The chi-square test was used to compare different CA-SSTIs in terms of categorical variables like diagnostic and treatment modalities. We divided the data into two groups based on the surgical procedure. A chi-square analysis was conducted to compare these two groups in terms of categorical variables.

Results: Out of the 241 patients, 51.9% were males and the mean age was 34.2 years. The most common CA-SSTIs were abscesses, infected ulcers, and cellulitis. Antibiotics were prescribed to 84.2% of patients. Amoxicillin + Clavulanate was the most frequently prescribed antibiotic. Out of the total, 128 (53.11%) patients received some type of surgical intervention. Surgical procedures were significantly associated with diabetes mellitus, heart disease, limitation of mobility, or recent antibiotic use. There was a significantly higher rate of prescription of any antibiotic and anti-methicillin-resistant *Staphylococcus aureus* (anti-MRSA) agents in the surgical procedure group. This group also saw a higher rate of oral antibiotics prescription, hospitalization, wound culture, and complete blood count.

Conclusion: This study shows a higher frequency of purulent infections in our ED. Antibiotics were prescribed more frequently for all infections. Surgical procedures like incision and drainage were much lower even in purulent infections. Furthermore, beta-lactam antibiotics like Amoxicillin-Clavulanate were commonly prescribed. Linezolid was the only systemic anti-MRSA agent prescribed. We suggest physicians should prescribe antibiotics appropriate to the local antibiograms and the latest guidelines.

## Introduction

Skin and soft tissue infections (SSTIs) are one of the most common diseases presented to the emergency department (ED) [1]. As of 2014, there were an estimated 29.7 SSTI-related emergency room visits per 1000 population in the ED of the United States [2].

Normally, many microorganisms colonize the skin without causing any harm. With any imbalance in the structural or functional protection of the skin, the pathogenic organisms spread in the layers of the skin, overgrow, and elicit acute or chronic inflammation [1]. This process is called infection. Some skin infections may result from a hematogenous spread of pathogens from a distant infection [1]. Most SSTIs are caused by streptococci and *Staphylococcus aureus* [3].

The United States food and drug authority classified SSTIs as uncomplicated and complicated infections [4,5]. Uncomplicated SSTIs include simple abscesses, cellulitis, impetigo, and furuncles. Complicated SSTIs include severe infections like necrotizing infections, infected burn wounds, infected open ulcers, and deep abscesses requiring major surgical intervention. It also includes infections in diabetic patients and immunocompromised patients [4].

Most simple SSTIs self-resolve. Large, complicated, and/or painful SSTIs require medical attention. Depending upon the severity, infections may need antibiotics and/or a surgical procedure like syringe aspiration, incision and drainage (I/D), debridement and drainage (D/D), or even amputation [6]. In an outpatient department and infectious disease setting, the focus is to provide specific antibiotics tailored to the sensitivity results. However, in the emergency room, physicians are mainly concerned with empiric therapy [7].

Based on the suspected source of infection, the SSTIs can be divided into two groups: Community-acquired: infections in non-hospitalized patients; and healthcare-associated: when an infection is acquired during or soon after hospitalization [8]. The healthcare-associated infections are considered a major complication, so they are frequently studied everywhere.

Community-acquired skin and soft tissue infections (CA-SSTIs) are relatively less studied. There is no study available on the management of CA-SSTIs in our population. The main objective of our study was to describe the frequency and distribution of CA-SSTIs, as well as medical and surgical treatments employed for these infections in our ED. This can then be used for further study to improve our practices under the current guidelines.

## Materials and methods

We conducted a descriptive cross-sectional study on patients presenting with CA-SSTIs to the ED of a tertiary care hospital in Peshawar, Pakistan. After approval from the hospital ethics and research board, we collected data in the ED from 1 September 2022 to 31 October 2022.

Through a consecutive sampling technique, we included all the patients, of any age or gender, that presented with an active SSTI. We excluded the patients who did not consent to data collection, who visited for a follow-up of a past infection, and/or who, by definition, had a healthcare-associated infection. Any infection that presents 48 hours after hospital admission, within three days after discharge, or within 30 days of surgery, was called a healthcare-associated infection [9].

After informed verbal consent, we collected the required data on a pre-designed proforma (Appendices). This included the patient’s biodata, baseline variables, comorbid conditions, diagnostic tests, and the treatment modalities used. A telephonic follow-up was conducted regarding the disease status after one and two weeks of the visit.

Data analysis

We collected data from 264 patients based on an anticipated frequency of 3.18% SSTIs in the ED and a 95% level of confidence [2]. Twenty-three patients were excluded due to deficient or incorrect information. The final analysis was carried out on 241 patients in Statistical Product and Service Solutions (SPSS) (IBM SPSS Statistics for Windows, Version 25.0, Armonk, NY).

Descriptive statistics were obtained for quantitative variables like age. Frequencies and percentages were derived for categorical variables including gender, age groups, clinical diagnosis, diagnostic tests, and treatment modalities used. The chi-square test was used to compare different SSTIs in terms of categorical variables like diagnostic modalities. A p-value of less than 0.05 was considered a statistically significant association.

We further divided the data into two groups based on the surgical procedure done: the surgical procedure group (those who underwent any surgical procedure including syringe aspiration, I/D, D/D, or amputation for a CA-SSTI), and the non-surgical procedure group. A chi-square analysis was conducted to compare these two groups in terms of categorical variables. A p-value of less the 0.05 was considered a statistically significant association.

## Results

**Figure 1:**
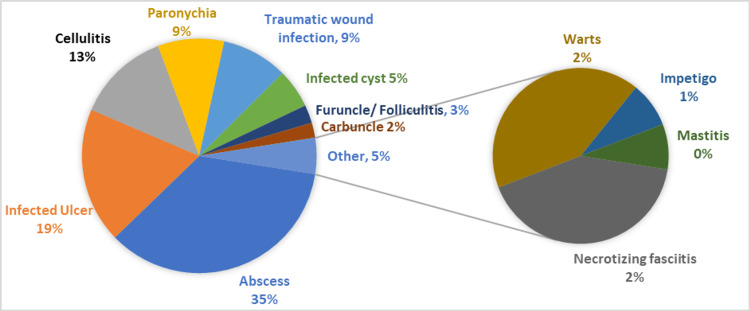
Distribution (in Percentages) of Different CA-SSTIs Presenting to the Emergency Department of Tertiary Care Hospital, Peshawar.

Out of the 241 patients, 51.9% were males and the mean ± SD age was 34.2 ± 18.1 (Table 1). The most common CA-SSTIs were abscesses (35.3%), infected ulcers (18.7%), and cellulitis (12.9%) (Figure 1).

**Table 1:** Demographic Distribution of the Sample for Patients Who Visited ED with CA-SSTI, September 2022 to October 2022. ED: emergency department CA-SSTI: community-acquired skin and soft tissue infections

Sample Size		241
Age (years)		
Mean (± standard deviation)		34.2 ± 18.1
Minimum		1 year
Maximum		80 years
	Frequency	Percentage
Age Group		
0-10 years	24	10%
11-20 years	33	13.7%
21-30 years	64	26.6%
31-40 years	36	14.9%
41-50 years	32	13.3%
51-60 years	39	16.2%
61-70 years	10	4.1%
71-80 years	3	1.2%
Gender		
Male	142	58.9%
Female	99	41.1%

**Figure 2:**
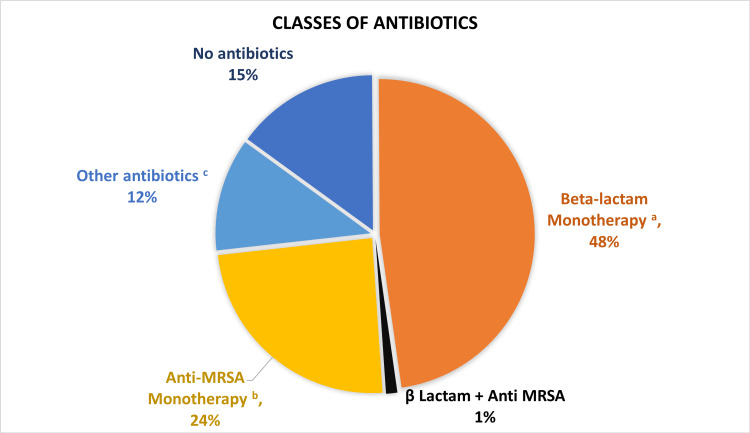
Different Classes of Antibiotics Prescribed for the Patients Presented to the ED with Skin and Soft Tissue infections. a: β Lactam antibiotics included Penicillins, Cephalosporins, and Carbapenems b: Anti-methicillin-resistant *Staphylococcus aureus* (anti-MRSA) included Linezolid and Mupirocin c: Others: Fusidic acid, Macrolides, Aminoglycosides, Quinolones, Rifampin, Polymyxin B, and Colistin ED: emergency department

**Figure 3:**
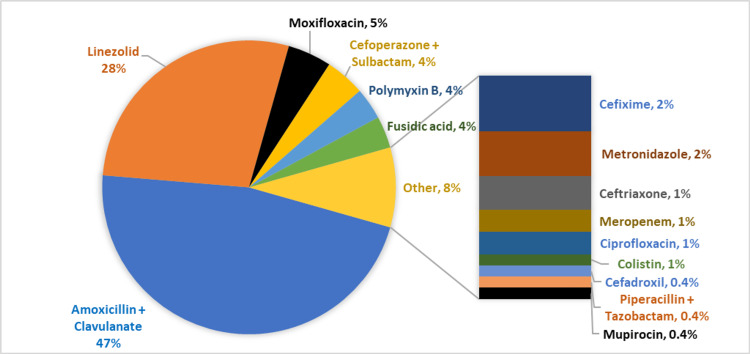
Different Types of Antibiotics Prescribed for the Patients Presented to the ED with Skin and Soft Tissue Infections. ED: emergency department

Antibiotics were prescribed to 84.2% of patients (Table 2). Amoxicillin + Clavulanate was the most common type of antibiotic prescribed followed by Linezolid, Moxifloxacin, and Cefoperazone + Sulbactam (Figures 2, 3).

**Table 2:** Frequencies and Percentages of the Different Diagnostic and Treatment Modalities Used. CBC: complete blood count

Variable	Frequency (n=241)	Percentage
Hospitalized	30	12.4%
Diagnostic modalities		
CBC ordered	62	25.7%
Wound culture ordered	35	14.5%
Blood culture ordered	2	0.8%
Treatment modalities		
Any antibiotic ordered	203	84.2%
Antibiotics only	83	34.4%
Surgical procedure ordered	128	53.1%
Surgical procedure only	8	3.3%
Antibiotics + Procedure	120	49.8%
No antibiotics	38	15.7%
No antibiotics or procedure	30	12.4%
Route of administration		
Oral	177	73.4%
Topical	16	6.6%
Parenteral	29	12%

Out of the total, 128 (53.11%) patients received some type of surgical intervention while 113 (46.8%) did not receive surgical intervention. Surgical procedures were significantly more common in patients with diabetes mellitus, heart disease, limitation of mobility, or recent antibiotic use. However, there was no significant difference between the two groups for other variables (Table 3).

**Table 3:** Association of the Surgical Procedures Group with the Non-surgical Procedure Group with Respect to Different Baseline Characteristics and Comorbid Conditions of Patients. All the associations were drawn using a two-sided chi-square test and the values are shown in percentages of the total procedures done or not done. a: p-value < 0.05 indicate significant association and are written in italic. b: CKD: chronic kidney disease c: CLD: chronic liver disease NOTE: The percentages may not add up to 100 due to rounding off.

Variables	Overall (n=241)	Surgical Procedure Done (n=128)	No Surgical Procedure (n=113)	p-value^a^
	%	%	%	
Gender				
Males	58.9%	59.4%	58.4%	0.879
Females	41.1%	40.6%	41.6%	
Other baseline variables				
Pregnant	1.2%	0.8%	1.8%	0.49
Lactating	3.3%	1.6%	5.3%	0.105
Systemic infection	49%	37%	0.371%	0.371
Sepsis	3.3%	3.9%	2.7%	0.588
Co-morbidities				
Diabetic	30.3%	35.9%	23.9%	0.042
CKD^b^	0.8%	1.6%	0%	0.182
CLD^c^	0.8%	0.8%	0.8%	0.929
Heart disease	4.6%	7.8%	0.9%	0.01
Limitation of mobility	11.2%	15.6%	6.2%	0.021
Injection drug abuse	0.4%	0.8%	0%	0.346
Recurrent skin infections	11.6%	14.1%	8.8%	0.208
Recent antibiotic use	40.2%	47.7%	31.9%	0.013

With respect to management, there was a significantly higher rate of prescription of any antibiotic and anti-methicillin-resistant *Staphylococcus aureus* (anti-MRSA) antibiotics in the surgical procedure group. We also saw a higher rate of oral antibiotics prescription, hospitalization, wound culture, and complete blood count (CBC) in this group (Table 4).

Although follow-up was included as a secondary objective of our study, only 129 patients responded to follow-up communication (53.3%). Using a univariate chi-square analysis on these 129 cases showed no statistically significant difference in improvement between the surgical and non-surgical groups (Table 4).

**Table 4:** Association of the Different Diagnostic and Therapeutic Modalities Used in the Patients who Underwent a Surgical Procedure for the CA-SSTIs in Comparison to Those who Received No Surgical Treatment at All. a: The association has been derived using a two-sided chi-square test, and the statistical significance corresponds to the p-value < .05. Significant values are italicized. b: Beta-lactam antibiotics: Penicillins, Cephalosporins, Carbapenems c: Anti-methicillin-resistant *Staphylococcus aureus* (anti-MRSA) antibiotics: Sulfonamides, Tetracyclines, Clindamycin, Vancomycin, Daptomycin, Tigecycline, Mupirocin d: Others: Macrolides, Aminoglycosides, Quinolones, Rifampin, Polymyxins e: CBC: complete blood count f: Follow-up was successful only in 129/241 patients (53.3%), and the chi-square analysis for follow-up was carried out only in these 129 cases. CA-SSTIs: community-acquired skin and soft tissue infections

Variables	Surgical Procedure Done (n=128)	No Surgical Procedure (n=113)	p-value^a^
	%	%	
Category of Antibiotics			
Any Antibiotics Prescribed	93.8	73.5	< .001
Beta-lactam monotherapy^b^	50	51.3	.837
Anti-MRSA monotherapy^c^	36.7	13.3	< .001
Beta lactam + Anti MRSA	1.6	0.9	.636
Other Antibiotics^d^	12.5	12.4	.979
No Antibiotics	6.3	26.5	< .001
Route of administration			
Topical Antibiotics	7	6.2	.795
Oral Antibiotics	80.5	65.5	.009
Parenteral Antibiotics	15.6	8	.068
Hospitalized	21.9	1.8	< .001
Laboratory work-up			
Wound Culture	22.7	5.3	< .001
CBC^e^	35.2	15	< .001
Blood culture	0.8	0.9	.929
Follow-up(n=129)^f^			
One week improvement	36.7	50	.136
Two weeks improvement	68.4	74	.493

Finally, we compared different CA-SSTIs in terms of the proportions of different diagnostic and treatment modalities using a univariate chi-square. We found a statistically significant association of the different SSTIs with wound culture (p<0.001), CBC (p<0.001), blood culture (p=0.014), and hospitalization (p<0.001). There was no significant difference in the antibiotic prescription and surgical procedure for different CA-SSTIs (Table 5).

**Table 5:** Association of Different Skin and Soft Tissue Infections with Different Diagnostic and Treatment Modalities Utilized. The Association Has Been Derived Using a Chi-square Test for Each Dependent Variable. a: p-value less than 0.05 is considered statistically significant and is written in italics b: CBC: Complete blood count

		Wound Culture Ordered *[p < 0.001] ^a^*	CBC^b^ *[p < 0.001]*	Blood Culture *[p = 0.014]*	Antibiotics Ordered [p = 0.53]	Surgical Procedure [p = 0.24]	Hospitalized *[p < 0.001]*
Clinical Diagnosis	Frequency (n)	%	%	%	%	%	%
Abscess	85	21.2%	24.7%	1.2%	84.7%	50.6%	8.2%
Infected Ulcer	45	17.8%	53.3%	0%	73.3%	64.4%%	24.4%
Cellulitis	31	0%	9.7%	0%	80.6%	38.7%	9.7%
Paronychia	22	0%	0%	0%	95.5%	45.5%	4.5%
Traumatic wound infection	22	0%	4.5%	0%	90.9%	59.1%	4.5%
Infected cyst	13	15.4%	30.8%	0%	100%	69.2%	7.7%
Furuncle/Folliculitis	6	16.7%	0%	0%	100%	33.3%	0%
Carbuncle	5	60%	80%	0%	100%	80%	40%
Necrotizing fasciitis	5	60%	100%	20%	80%	80%	80%
Warts	5	0%	0%	0%	40%	40%	0%
Impetigo	1	0%	0%	0%	100%	0%	0%
Mastitis	1	0%	0%	0%	100%	0%	0%
Total	241	14.5%	25.7%	0.8%	84.2%	53.1%	12.4%

## Discussion

SSTIs are common presentations in ED [10]. Mistry et al. reported that 51% of the SSTI patients treated in the ED were males and 49% were females [6]. In addition, 57% of their patients belonged to the 18-49 years age group. Our study showed 59% male patients versus 41% females. Most of our patients were in the 21-30 years age group (26.6%). The most common CA-SSTIs were abscesses, infected ulcers, and cellulitis.

Around half (53.1%) of our patients received some sort of surgical procedure and 84% received an antibiotic. The surgical procedure was strongly associated with diabetes, heart disease, limitation of mobility, and recent antibiotic use as these factors are associated with severe purulent infections.

Among the purulent infections, a surgical procedure was performed in 50.6% of abscesses, 80% of carbuncles, 33% of furuncles, 69% of infected cysts, and 80% of necrotizing fasciitis patients. The rate of antibiotic prescription was much higher in all these infections. Mistry et al. reported I/D in 27% of patients presenting with an SSTI to the ED with an antibiotic prescription rate of 85% overall. Like other hospitals, the instinctive practice of antibiotics prescription more than I/D exists in our setting as well.

Diagnosis of these infections is mostly clinical [1]. Guidelines suggest that CBC and wound culture should be ordered for all complicated SSTIs, and for those with sepsis should be ordered for blood culture as well [11]. These tests can be avoided in uncomplicated infections [1]. In our study, wound culture was ordered in 14% of patients, more commonly in the surgical procedure group (22.7%). CBC was also strongly associated with surgical procedures (p<0.001). Mistry et al. reported similar results with wound culture in 16% of patients, more common in those receiving an I/D. However, this study reported a higher rate of CBC and blood culture in the non-surgical group. Kamath et al. reported wound culture in all the patients (100%) undergoing I/D for an SSTI [11].

Although financial affordability is a huge limitation in our setting, wound culture is still strongly advisable in patients undergoing a surgical procedure for an SSTI. This will promote antibiotic stewardship and reduce antibiotic resistance.

Our study reported a 12.4% hospitalization, more commonly in necrotizing fasciitis, carbuncles, and infected ulcers. It was also strongly associated with surgical procedures (21.9%). Hospitalization for SSTI is associated with larger lesions, fever, and comorbidities [12]. Mistry et al. also reported a strong association between hospitalization and I/D. In compliance with the guidelines, our emergency-based hospitalization is very selective and reserved only for severe conditions as reported [11].

In a large multicenter study from the United States, Fritz et al. reported that about 58.6% of patients received anti-MRSA drugs and only 35.1% beta-lactam drugs for ambulatory patients with SSTIs [10]. The most common antibiotics were trimethoprim-sulfamethoxazole (TMP-SMX), cephalexin, clindamycin, and doxycycline [10]. Antibiotics were prescribed to 84% of our patients. The surgical procedure group was more likely to receive any antibiotics (94%) and anti-MRSA antibiotics (36.7%). In a large multicenter study from the United States, Mistry et al. reported no difference in the antibiotic prescription rate but showed that anti-MRSA drugs were more commonly prescribed to those undergoing an I/D. Guidelines by the Infectious Disease Society of America (IDSA) recommend I/D or D/D along with anti-MRSA agents as the first-line treatment for purulent infections [11].

Only 25% of our patients received anti-MRSA drugs and 49% received beta-lactam antibiotics. The most common antibiotics were Amoxicillin-Clavulanate and Linezolid. Although MRSA is a significant problem in our population [13], we are still relying on beta-lactam antibiotics mostly. This can be attributed to the lower awareness of the physicians on the updated local antibiogram and current international guidelines.

According to a recent local study, highly sensitive anti-MRSA antibiotics include Fusidic acid, Teicoplanin, Chloramphenicol, Doxycycline, and Linezolid [14]. Another recent antibiogram from our population reported a high susceptibility of MRSA isolates to Vancomycin and Amikacin (94.4%), followed by Teicoplanin, Doxycycline, Ciprofloxacin, Cefotaxime, Tigecycline, Clindamycin, and Linezolid [15]. While we have all these options available against MRSA infection, only Linezolid was prescribed as a systemic anti-MRSA agent.

Although Linezolid is highly effective against MRSA and other staphylococci [16], the unchecked generous use of Linezolid as empiric therapy might result in the disastrous growth of extended drug-resistant staphylococci. Further study is required on the anti-MRSA antibiotics prescription in comparison to the local antibiogram. Awareness is required to promote the use of other first-line anti-MRSA drugs like clindamycin, TMP-SMX, doxycycline, vancomycin, etc. rather than only Linezolid.

Although our study showed no difference in the outcomes in both groups, it is difficult to discuss this aspect due to the loss of follow-up.

Limitations

This prospective study gives a detailed insight into the burden of a prevalent complaint in our ED. It also describes in detail the current practices in our population. However, it has a few limitations: (1) loss of follow-up in about half the patients, (2) no microbiologic comparison, (3) a relatively small sample from only one center, and (4) multivariable regression analysis was not performed. Although a multivariate analysis is ideal to evaluate the association of multiple independent variables with a dependent variable, this type of analysis gives erroneous results in studies with a relatively small sample size [17].

## Conclusions

This study shows a higher frequency of purulent infections among the SSTIs presenting to our ED. Antibiotics were prescribed more frequently for all infections, though surgical procedures like incision and drainage were much lower even in purulent infections. Furthermore, beta-lactam antibiotics like Amoxicillin-Clavulanate were commonly prescribed. Linezolid was the only systemic anti-MRSA agent prescribed.

We suggest physicians should prescribe antibiotics appropriate to the local antibiograms and the latest guidelines. We also recommend further studies on these infections in comparison to the microbiologic studies.

## Figures and Tables

**Figure 4:**
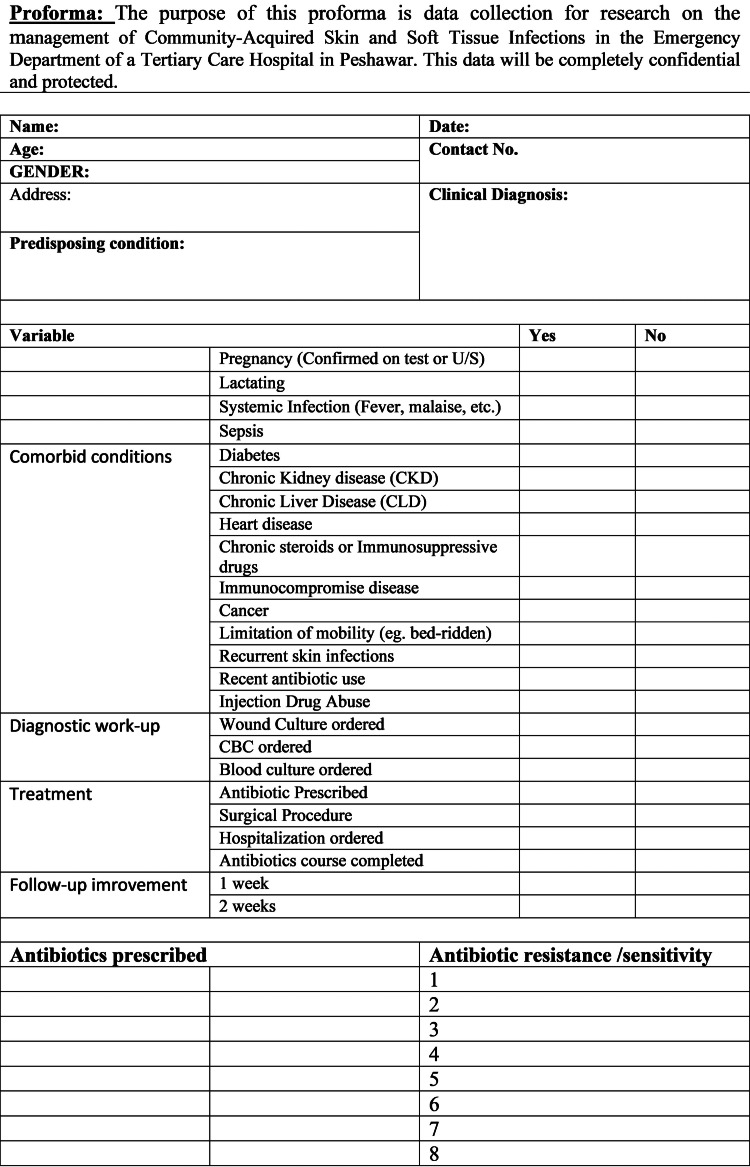
Proforma for Data Collection.
